# Supplementary material for: The effect of COVID-19 and socioeconomic inequalities on emergency department accesses for psychiatric conditions
Source: PLoS One. 2026 Jul 21;21(7):e0324305. doi: 10.1371/journal.pone.0324305 (PMC13387567; doi:10.1371/journal.pone.0324305)
Supplement: S1 Table — (PDF) [file pone.0324305.s001.pdf]

S1 Table

FMHEA characteristics	Mean (per month)	SD	Trend pre-COVID-19	COVID-19 immediate effect	Trend post-COVID-19
			IRR (95%CI)	IRR (95%CI)	IRR (95%CI)
**Total**	1182.5	269.2	**0.992 (0.988-0.997)**	**0.636 (0.481-0.840)**	**1.018 (1.001-1.036)**
**Sex**					
Males	492.6	102.2	**0.992 (0.988-0.997)**	**0.684 (0.534-0.878)**	**1.016 (1.001-1.032)**
Females	690.0	170.5	**0.993 (0.988-0.997)**	**0.602 (0.431-0.840)**	1.020 (0.999-1.041)
-	-	-	-	-	-
**Age class**					
≤24	230.4	51.3	0.998 (0.994-1.003)	**0.559 (0.412-0.758)**	**1.029 (1.028-1.048)**
25-34	146.5	42.6	**0.988 (0.980-0.995)**	**0.637 (0.446-0.910)**	1.019 (0.996-1.043)
35-64	493.8	120.8	**0.991 (0.985-0.996)**	**0.672 (0.506-0.893)**	1.012 (0.994-1.030)
65-74	115.2	23.6	**0.993 (0.989-0.998)**	**0.697 (0.530-0.915)**	**1.021 (1.004-1.038)**
75-84	134.3	31.0	0.994 (0.987-1.000)	**0.664 (0.499-0.885)**	**1.020 (1.003-1.037)**
85+	62.3	20.3	**0.993 (0.990-0.996)**	**0.502 (0.423-0.597)**	**1.015 (1.005-1.026)**
-	-	-	-	-	-

**Deprivation level (quintiles)**					
Low	217.5	50.8	**0.993 (0.987-0.999)**	**0.592 (0.444-0.790)**	**1.031 (1.012-1.049)**
Middle-low	267.5	60.1	**0.990 (0.986-0.995)**	**0.654 (0.502-0.851)**	**1.020 (1.004-1.037)**
Middle	251.7	58.5	**0.994 (0.989-0.998)**	**0.619 (0.461-0.832)**	1.018 (0.999-1.037)
Middle- high	178.8	43.9	**0.993 (0.987-0.998)**	**0.659 (0.485-0.895)**	1.013 (0.993-1.032)
High	122.7	36.9	**0.992 (0.984-0.999)**	0.694 (0.470-1.027)	0.999 (0.976-1.023)
-	-	-	-	-	-
**Citizenship**					
Italians+HDCs	1050.5	242.3	**0.993 (0.989-0.997)**	**0.624 (0.473-0.824)**	**1.019 (1.001-1.037)**
Immigrants from HMPCs	117.9	24.3	**0.991 (0.986-0.997)**	**0.736 (0.580-0.935)**	1.013 (0.999-1.028)
-	-	-	-	-	-
**Group of diagnoses**					
Delirium/Mental Confusion	125.8	19.1	1.001 (0.998-1.005)	**0.811 (0.699-0.941)**	1.006 (0.997-1.014)
Dependencies	75.5	23.5	1.017 (0.990-1.044)	**0.605 (0.386-0.948)**	1.011 (0.985-1.037)
Eating Disorders	3.4	2.3	1.018 (0.978-1.060)	1.023 (0.517-2.027)	1.008 (0.971-1.046)

Anxiety Disorders	520.2	147.4	**0.991 (0.985-0.996)**	**0.658 (0.467-0.928)**	1.007 (0.985-1.030)
Mood Disorders	95.5	28.1	**0.988 (0.982-0.994)**	**0.681 (0.516-0.898)**	1.008 (0.991-1.026)
Personality Disorders	9.2	5.7	**0.947 (0.930-0.963)**	1.186 (0.841-1.673)	0.999 (0.989-1.009)
Dissociation	3.8	1.9	**0.979 (0.968-0.990)**	0.894 (0.565-1.414)	1.009 (0.975-1.043)
Unspecified Psychoses	102.2	17.0	0.998 (0.993-1.002)	0.916 (0.799-1.049)	1.001 (0.991-1.010)
Adjustment Disorders	14.2	5.0	0.995 (0.987-1.004)	**0.651 (0.472-0.898)**	1.014 (0.996-1.032)
Post-Traumatic Stress and related Disorders	25.4	8.2	0.997 (0.989-1.005)	**0.604 (0.411-0.890)**	1.007 (0.981-1.035)
Somatization	144.6	54.9	0.991 (0.983-1.000)	0.389 (0.269-0.562)	1.031 (1.009-1.054)
Schizophrenic Spectrum	5.8	2.6	**0.976 (0.972-0.980)**	**1.207 (1.071-1.360)**	**0.981 (0.971-0.990)**
-	-	-			
