# Supplementary figures and images for: The effect of COVID-19 and socioeconomic inequalities on emergency department accesses for psychiatric conditions
Source: PLoS One. 2026 Jul 21;21(7):e0324305. doi: 10.1371/journal.pone.0324305 (PMC13387567; doi:10.1371/journal.pone.0324305)

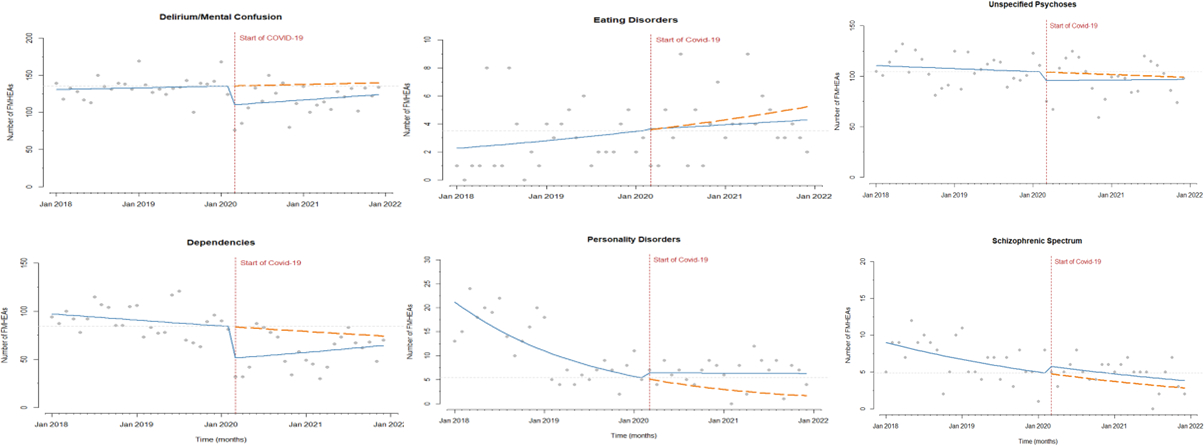

Supplement: S1 Fig — Observed monthly accesses are shown for each diagnosis. Vertical dashed line: introduction of restrictions. Continuous line: trend over the years. Dashed line: counterfactual scenario. Horizontal dashed line: pre-pandemic level. (TIF) [file pone.0324305.s002.tif]

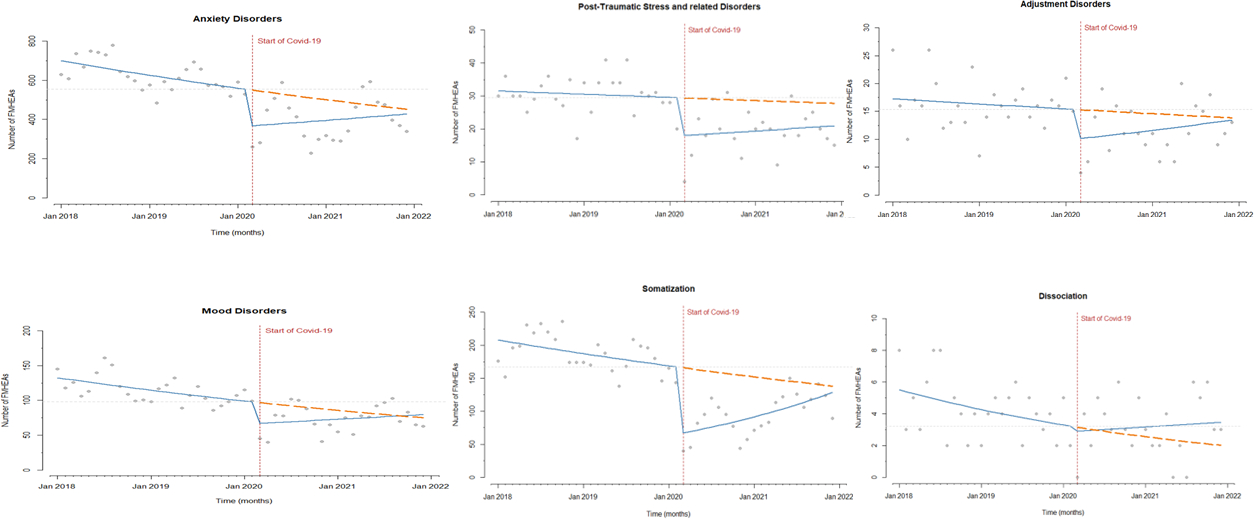

Supplement: S2 Fig — Observed monthly accesses are shown for each diagnosis. Vertical dashed line: introduction of restrictions. Continuous line: trend over the years. Dashed line: counterfactual scenario. Horizontal dashed line: pre-pandemic level. (TIF) [file pone.0324305.s003.tif]
