# Supplementary material for: The effect of COVID-19 and socioeconomic inequalities on emergency department accesses for psychiatric conditions
Source: PLoS One. 2026 Jul 21;21(7):e0324305. doi: 10.1371/journal.pone.0324305 (PMC13387567; doi:10.1371/journal.pone.0324305)
Supplement: Peer review history

# Peer review: Decision Letter 0

**DOI:** 10.1371/journal.pone.0324305.r001

**Authors:** Paul Obeng

18 May 2026

PONE-D-25-21941

The effect of COVID-19 and socioeconomic inequalities on emergency department accesses for psychiatric conditions

PLOS One

Dear Dr. SCHEPISI,

Thank you for submitting your manuscript to PLOS ONE. After careful consideration, we feel that it has merit but does not fully meet PLOS ONE’s publication criteria as it currently stands. Therefore, we invite you to submit a revised version of the manuscript that addresses the points raised during the review process.

Please submit your revised manuscript by Jul 02 2026 11:59PM. If you will need more time than this to complete your revisions, please reply to this message or contact the journal office at plosone@plos.org. When you're ready to submit your revision, log on to https://www.editorialmanager.com/pone/ and select the 'Submissions Needing Revision' folder to locate your manuscript file.

Please include the following items when submitting your revised manuscript:

- A letter that responds to each point raised by the academic editor and reviewer(s). You should upload this letter as a separate file labeled 'Response to Reviewers'.
- A marked-up copy of your manuscript that highlights changes made to the original version. You should upload this as a separate file labeled 'Revised Manuscript with Track Changes'.
- An unmarked version of your revised paper without tracked changes. You should upload this as a separate file labeled 'Manuscript'.

If you would like to make changes to your financial disclosure, please include your updated statement in your cover letter. Guidelines for resubmitting your figure files are available below the reviewer comments at the end of this letter.

If applicable, we recommend that you deposit your laboratory protocols in protocols.io to enhance the reproducibility of your results. Protocols.io assigns your protocol its own identifier (DOI) so that it can be cited independently in the future. For instructions see: https://journals.plos.org/plosone/s/submission-guidelines#loc-laboratory-protocols. Additionally, PLOS ONE offers an option for publishing peer-reviewed Lab Protocol articles, which describe protocols hosted on protocols.io. Read more information on sharing protocols at https://plos.org/protocols?utm_medium=editorial-email&utm_source=authorletters&utm_campaign=protocols.

As the corresponding author, your ORCID iD is verified in the submission system and will appear in the published article. PLOS supports the use of ORCID, and we encourage all coauthors to register for an ORCID iD and use it as well. Please encourage your coauthors to verify their ORCID iD within the submission system before final acceptance, as unverified ORCID iDs will not appear in the published article. *Only*  the individual author can complete the verification step; PLOS staff *cannot*  verify ORCID iDs on behalf of authors.

We look forward to receiving your revised manuscript.

Kind regards,

Paul Obeng, MEd, MSc., M.Phil.

Academic Editor

PLOS One

Journal Requirements:

When submitting your revision, we need you to address these additional requirements.

1.Please ensure that your manuscript meets PLOS ONE's style requirements, including those for file naming. The PLOS ONE style templates can be found at

https://journals.plos.org/plosone/s/file?id=wjVg/PLOSOne_formatting_sample_main_body.pdf and

https://journals.plos.org/plosone/s/file?id=ba62/PLOSOne_formatting_sample_title_authors_affiliations.pdf

2. In the online submission form you indicate that your data is not available for proprietary reasons and have provided a contact point for accessing this data. Please note that your current contact point is a co-author on this manuscript. According to our Data Policy, the contact point must not be an author on the manuscript and must be an institutional contact, ideally not an individual. Please revise your data statement to a non-author institutional point of contact, such as a data access or ethics committee, and send this to us via return email. Please also include contact information for the third party organization, and please include the full citation of where the data can be found.

3. Please include captions for your Supporting Information files at the end of your manuscript, and update any in-text citations to match accordingly. Please see our Supporting Information guidelines for more information: http://journals.plos.org/plosone/s/supporting-information.

4. If the reviewer comments include a recommendation to cite specific previously published works, please review and evaluate these publications to determine whether they are relevant and should be cited. There is no requirement to cite these works unless the editor has indicated otherwise.

5. Please review your reference list to ensure that it is complete and correct. If you have cited papers that have been retracted, please include the rationale for doing so in the manuscript text, or remove these references and replace them with relevant current references. Any changes to the reference list should be mentioned in the rebuttal letter that accompanies your revised manuscript. If you need to cite a retracted article, indicate the article’s retracted status in the References list and also include a citation and full reference for the retraction notice.

[Note: HTML markup is below. Please do not edit.]

Reviewers' comments:

Reviewer's Responses to Questions

**Comments to the Author**

1. Is the manuscript technically sound, and do the data support the conclusions?

The manuscript must describe a technically sound piece of scientific research with data that supports the conclusions. Experiments must have been conducted rigorously, with appropriate controls, replication, and sample sizes. The conclusions must be drawn appropriately based on the data presented.

Reviewer #1: Yes

Reviewer #2: Yes

**********

2. Has the statistical analysis been performed appropriately and rigorously?

Reviewer #1: Yes

Reviewer #2: Yes

**********

3. Have the authors made all data underlying the findings in their manuscript fully available?

The PLOS Data policy requires authors to make all data underlying the findings described in their manuscript fully available without restriction, with rare exception (please refer to the Data Availability Statement in the manuscript PDF file). The data should be provided as part of the manuscript or its supporting information, or deposited to a public repository. For example, in addition to summary statistics, the data points behind means, medians and variance measures should be available. If there are restrictions on publicly sharing data—e.g. participant privacy or use of data from a third party—those must be specified.

Reviewer #1: Yes

Reviewer #2: No

**********

4. Is the manuscript presented in an intelligible fashion and written in standard English?

PLOS ONE does not copyedit accepted manuscripts, so the language in submitted articles must be clear, correct, and unambiguous. Any typographical or grammatical errors should be corrected at revision, so please note any specific errors here.

Reviewer #1: Yes

Reviewer #2: Yes

**********

5. Review Comments to the Author

Please use the space provided to explain your answers to the questions above. You may also include additional comments for the author, including concerns about dual publication, research ethics, or publication ethics. (Please upload your review as an attachment if it exceeds 20,000 characters)

Reviewer #1: This is a good and well prepared manuscript.

However I have two suggestions.

1. The authors should please state the study design. It's not well stated in the method. One is left to extrapolate it in the method description or results. It should be stated clearly so that beginner researchers can follow.

2. The study period was divided into 2. Please why was the second period 1 year and the 1st 2 years. This is a potential source of bias and a limitation too. Please there has to be an explanation to defend it in the method or in limitation section.

Reviewer #2: I would like to acknowledge that this paper has valuable findings about the effect of COVID-19 and socioeconomic inequalities on emergency department accesses for psychiatric conditions. The paper had sound methodology it, however needs some major clarifications to improve on it's strength and clarity.

Generally, The translation was done well but had some confusing misinterpretations, however, this can also be improved by adding some definitions.

Abstract

The paper mentions a population based longitudinal open cohort of residents and NHS beneficiaries, was the number 5,159,365 the total number of people in the 3 large Italian areas >10 years? Or Was it the total number of NHS beneficiaries?

To make the abstract much easier to follow could you please defined the words NHS and FMHEA at first use, i struggled with these and only found them under the methods. Defining them early could improve the flow of the write up.

In the results of the abstract, the authors mention that the rate of FMHEA was higher among the highly deprived, which seemed different from what was observed in the results at least according to what was understood by the deprivation levels. Does this mean the highly deprived had higher access to emergency department for psychiatric conditions? It seems that results are indicating that. Lowest deprived vs highly deprived definitions.

The author needs to add a proper description of the levels of deprivation, for example does higher deprivation mean those in poor Socioeconomic status and vice versa? If so, then our previous statement above needs revision.

furthermore, the categorization of the deprivation level, middle low, middle , middle high seemed confusing and could cause collinearity in the data.

Under the methods section of the paper, the study setting lacked some clarity, i.e. the three centers Tuscany Regional Health Agency (RHA),the Bergamo Local Health Authority (LHA),and Rome 2 LHA are they medical facilities? regions?

For access of the data from Municipal registries and general practitioner registries, did the participants consent to access of this data? Did you obtain a waiver of consent?

Please clarify how you ensured that the cases you selected were incident cases without any prior access for any condition within the two years. Did you have access to the selected participants medical histories?

Using a census tract as proxy for individual level social economic status is inaccurate, might be a good proxy for neighborhood SES,this needs to be added in the limitations

Results section

Table 5 is very unclear and hard to follow, i recommend some improvements in the presentations either eliminate some of the variables that were not significant.

Lin2 277 in the discussion section ,the incidence rate of ED accesses seems to be 26% & not 29% as reported, please cross check.

Overall, The study has value. So many assumptions were made that do not make the study clear, e.g Participants were followed up for a period and those who had ED access (FMHEA) were analysed, were there some individuals who had mental illnesses that did not have access to the ED?

Another observation is that the results indicate modest increases and declines with very small effect sizes. The results therefore should be interpreted with caution.

**********

6. PLOS authors have the option to publish the peer review history of their article (what does this mean?). If published, this will include your full peer review and any attached files.

If you choose “no”, your identity will remain anonymous but your review may still be made public.

**Do you want your identity to be public for this peer review?**  For information about this choice, including consent withdrawal, please see our Privacy Policy.

Reviewer #1: **Yes:**  Noted

Reviewer #2: No

**********

[NOTE: If reviewer comments were submitted as an attachment file, they will be attached to this email and accessible via the submission site. Please log into your account, locate the manuscript record, and check for the action link "View Attachments". If this link does not appear, there are no attachment files.]

To ensure your figures meet our technical requirements, please review our figure guidelines: https://journals.plos.org/plosone/s/figures

You may also use PLOS’s free figure tool, NAAS, to help you prepare publication quality figures: https://journals.plos.org/plosone/s/figures#loc-tools-for-figure-preparation.

NAAS will assess whether your figures meet our technical requirements by comparing each figure against our figure specifications.

# Author comment: Author response to Decision Letter 1

**DOI:** 10.1371/journal.pone.0324305.r002

20 Jun 2026

EDITOR’S COMMENTS

When submitting your revision, we need you to address these additional requirements.

1.Please ensure that your manuscript meets PLOS ONE's style requirements, including those for file naming. The PLOS ONE style templates can be found at

https://journals.plos.org/plosone/s/file?id=wjVg/PLOSOne_formatting_sample_main_body.pdf and

https://journals.plos.org/plosone/s/file?id=ba62/PLOSOne_formatting_sample_title_authors_affiliations.pdf

We addressed this point by doing the appropriate corrections

2. In the online submission form you indicate that your data is not available for proprietary reasons and have provided a contact point for accessing this data. Please note that your current contact point is a co-author on this manuscript. According to our Data Policy, the contact point must not be an author on the manuscript and must be an institutional contact, ideally not an individual. Please revise your data statement to a non-author institutional point of contact, such as a data access or ethics committee, and send this to us via return email. Please also include contact information for the third party organization, and please include the full citation of where the data can be found.

Data cannot be shared publicly because of data sharing legal restrictions on individual records. The anonymized dataset may be requested only for research or study purposes by applying to INMP (inmp@pec.inmp.it).

3. Please include captions for your Supporting Information files at the end of your manuscript, and update any in-text citations to match accordingly. Please see our Supporting Information guidelines for more information: http://journals.plos.org/plosone/s/supporting-information.

Captions have been included at the end of the manuscript and in text citations have been updated accordingly

4. If the reviewer comments include a recommendation to cite specific previously published works, please review and evaluate these publications to determine whether they are relevant and should be cited. There is no requirement to cite these works unless the editor has indicated otherwise.

Reviewers did not suggest adding further publications

5. Please review your reference list to ensure that it is complete and correct. If you have cited papers that have been retracted, please include the rationale for doing so in the manuscript text, or remove these references and replace them with relevant current references. Any changes to the reference list should be mentioned in the rebuttal letter that accompanies your revised manuscript. If you need to cite a retracted article, indicate the article’s retracted status in the References list and also include a citation and full reference for the retraction notice.

We carefully reviewed the reference list and three duplicate citations were identified and the corresponding articles were removed . Also, we verified the status of Jacob et al. (Health Policy, 2024;150:105168), for which an erratum was subsequently published. The correction concerns the omission of funding and data acknowledgement statements in the final published version and does not affect the study design, methods, analyses, results, or conclusions. We found no retraction notice or expression of concern associated with this publication so we retained it in the manuscript.

REVIEWERS’ COMMENTS

Reviewer #1:

This is a good and well-prepared manuscript.

However, I have two suggestions.

1. The authors should please state the study design. It's not well stated in the method. One is left to extrapolate it in the method description or results. It should be stated clearly so that beginner researchers can follow.

We have added an explicit citation of the study design both in the abstract and in the methods section.

2. The study period was divided into 2. Please why was the second period 1 year and the 1st 2 years. This is a potential source of bias and a limitation too. Please there has to be an explanation to defend it in the method or in limitation section.

In order to compare post- to pre-COVID trends the study period was divided into two phases based on the WHO declaration of pandemic outbreak and the beginning of the lockdown in Italy (March 2020). We understand that this may be a potential source of bias. However, incidence rate ratios (IRRs), by incorporating person-time in the denominator, inherently adjust for differences in follow-up duration between periods and ensure appropriate comparability. Lastly, the enrollment of the cohort has been carried on until December 2024, thus allowing to extend the post-pandemic observation.

Reviewer #2:

I would like to acknowledge that this paper has valuable findings about the effect of COVID-19 and socioeconomic inequalities on emergency department accesses for psychiatric conditions. The paper had sound methodology it, however needs some major clarifications to improve on it's strength and clarity.

Generally, The translation was done well but had some confusing misinterpretations, however, this can also be improved by adding some definitions.

We thank the reviewer for the comments, and we have addressed all the points raised as follows

Abstract

The paper mentions a population based longitudinal open cohort of residents and NHS beneficiaries, was the number 5,159,365 the total number of people in the 3 large Italian areas >10 years? Or was it the total number of NHS beneficiaries?

The number 5,167,043 refers to all residents for at least two years in one of three catchment areas, aged ≥ 10 years and assisted by a National Health Service (NHS) general practitioner (GP) of the area of residence.

To make the abstract much easier to follow could you please defined the words NHS and FMHEA at first use, I struggled with these and only found them under the methods. Defining them early could improve the flow of the write up.

Definitions of both the terms NHS and FMHEA have been included in the abstract.

In the results of the abstract, the authors mention that the rate of FMHEA was higher among the highly deprived, which seemed different from what was observed in the results at least according to what was understood by the deprivation levels. Does this mean the highly deprived had higher access to emergency department for psychiatric conditions? It seems that results are indicating that. Lowest deprived vs highly deprived definitions.

Incidence rates of Emergency accesses are higher in the highly deprived with respect to middle-high, middle-low and low levels of deprivation, both pre- and post-COVID (table 3). This means that the most deprived individuals (i.e. those suffering the harsh socioeconomic conditions) use the Emergency Department for psychiatric conditions more frequently than those in the other deprivation groups, both before and after COVID. Accordingly, when we measure IRR taking low deprivation as a reference, the pre-COVID IRR of the highly deprived is the highest among deprivation levels (1.28). We then performed an interaction analysis between COVID and deprivation level: it was shown that COVID had the greatest impact in terms of reduction of FMHEAs among the highly deprived. We acknowledge that definitions of low and high deprivation may seem confusing, therefore we added a sentence that clarifies the concept in the methods section.

The author needs to add a proper description of the levels of deprivation, for example does higher deprivation mean those in poor Socioeconomic status and vice versa? If so, then our previous statement above needs revision. Furthermore, the categorization of the deprivation level, middle low, middle , middle high seemed confusing and could cause collinearity in the data.

Regarding the categorization (low, middle-low, middle, middle-high, high), we acknowledge that the terminology may appear unclear. These categories were derived from the quintiles of the continuous indicator, which are commonly used to capture gradients in socioeconomic status rather than a simple binary classification. Importantly, the deprivation variable was included in the models as a single categorical variable. Therefore, issues of collinearity between its levels do not arise, as the categories are mutually exclusive and represent different strata of the same construct.

Under the methods section of the paper, the study setting lacked some clarity, i.e. the three centers Tuscany Regional Health Agency (RHA), the Bergamo Local Health Authority (LHA),and Rome 2 LHA are they medical facilities? regions?

We acknowledge that description of study settings lacks clarity. Therefore, we added a more detailed description of RHA and LHAs in the methods section.

For access of the data from Municipal registries and general practitioner registries, did the participants consent to access of this data? Did you obtain a waiver of consent?

Under Italian legislation, written consent is required for medical treatments. Conversely, recording of data on municipal and health registries is mandatory by law, and privacy must be granted. The study protocol was approved by the ethics committee of the Italian National Institute of Health and by Data Protection Officers of each participating center.

Please clarify how you ensured that the cases you selected were incident cases without any prior access for any condition within the two years. Did you have access to the selected participants medical histories?

We thank the reviewer for the comment, as we agree that definition of incident access was unclear. We have considered as incident accesses those cases where no other access in the two years before the observation was reported by medical registries, through which any access within the NHS is recorded and medical history can be retrieved. We modified the text in the outcome section of the methods.

Using a census tract as proxy for individual level social economic status is inaccurate, might be a good proxy for neighborhood SES, this needs to be added in the limitations

We raised the issue of using the census tract in the limits section. Despite the risk of residual ecological bias, we believe that, as a census tract refers to a very small area, it may be considered a good, or at least acceptable, proxy of socioeconomic status.

Results section

Table 5 is very unclear and hard to follow, I recommend some improvements in the presentations either eliminate some of the variables that were not significant.

We thank the reviewer for this suggestion. Given that monthly admission data may be more effectively conveyed through graphical representations, we have included in the main manuscript all figures depicting the effects of the covariates on monthly admission trends, while Table 5 has been relocated to the Supplementary Materials.

Line 277 in the discussion section, the incidence rate of ED accesses seems to be 26% & not 29% as reported, please cross check.

Incidence rate is reduced by 26%, value has been corrected in the discussion.

Overall, the study has value. So many assumptions were made that do not make the study clear, e.g Participants were followed up for a period and those who had ED access (FMHEA) were analysed, were there some individuals who had mental illnesses that did not have access to the ED?

This study is part of the larger COMEH project, which specifically investigated the impact of COVID on mental health through the analysis of mental healthcare utilization patterns. Within this broader aim, the main and specific outcome of the current study is incident accesses to the Emergency Department for psychiatric conditions. In previous studies, we have investigated the impact of COVID 19 on all mental healthcare services and drug prescriptions (Petrelli et al. 2025), and on psychiatric hospitalizations (Aragona et al. 2025).

Another observation is that the results indicate modest increases and declines with very small effect sizes. The results therefore should be interpreted with caution.

For some of the covariates, the effect size was modest as reflected by the IRR magnitude and when considered relative to the large sample size. We added this consideration in the limit section. However, it is to note that in the case of the effect of high deprivation, IRR shows a strong impact of COVID in this group, thus supporting the role of socioeconomic inequalities in shaping mental healthcare utilization patterns.

Attachment Submitted filename: ResponseToReviewers.docx

# Peer review: Decision Letter 1

**DOI:** 10.1371/journal.pone.0324305.r003

**Authors:** Paul Obeng

1 Jul 2026

The effect of COVID-19 and socioeconomic inequalities on emergency department accesses for psychiatric conditions

PONE-D-25-21941R1

Dear Dr. Schepisi,

We are pleased to inform you that your manuscript has been judged scientifically suitable for publication and will be formally accepted for publication once it meets all outstanding technical requirements.

Within one week, you’ll receive an e-mail detailing the required amendments. When these have been addressed, you’ll receive a formal acceptance letter, and your manuscript will be scheduled for publication.

An invoice will be generated when your article is formally accepted. Please note, if your institution has a publishing partnership with PLOS and your article meets the relevant criteria, all or part of your publication costs will be covered. Please make sure your user information is up-to-date by logging into Editorial Manager at Editorial Manager® and clicking the ‘Update My Information' link at the top of the page. For questions related to billing, please contact billing support.

If your institution or institutions have a press office, please notify them about your upcoming paper to help maximise its impact. If they’ll be preparing press materials, please inform our press team as soon as possible -- no later than 48 hours after receiving the formal acceptance. Your manuscript will remain under strict press embargo until 2 pm Eastern Time on the date of publication. For more information, please contact onepress@plos.org.

Kind regards,

Paul Obeng, MEd, MSc., M.Phil.

Academic Editor

PLOS One

Reviewers' comments:

Reviewer's Responses to Questions

**Comments to the Author**

1. If the authors have adequately addressed your comments raised in a previous round of review and you feel that this manuscript is now acceptable for publication, you may indicate that here to bypass the “Comments to the Author” section, enter your conflict of interest statement in the “Confidential to Editor” section, and submit your "Accept" recommendation.

Reviewer #2: All comments have been addressed

# Editor report: Acceptance letter

**DOI:** 10.1371/journal.pone.0324305.r004

**Authors:** Paul Obeng

PONE-D-25-21941R1

PLOS One

Dear Dr. SCHEPISI,

I'm pleased to inform you that your manuscript has been deemed suitable for publication in PLOS One. Congratulations! Your manuscript is now being handed over to our production team.

At this stage, our production department will prepare your paper for publication. This includes ensuring the following:

* All references, tables, and figures are properly cited

* All relevant supporting information is included in the manuscript submission,

* There are no issues that prevent the paper from being properly typeset

You will receive further instructions from the production team, including instructions on how to review your proof when it is ready. Please keep in mind that we are working through a large volume of accepted articles, so please give us a few days to review your paper and let you know the next and final steps.

Lastly, if your institution or institutions have a press office, please let them know about your upcoming paper now to help maximize its impact. If they'll be preparing press materials, please inform our press team within the next 48 hours. Your manuscript will remain under strict press embargo until 2 pm Eastern Time on the date of publication. For more information, please contact onepress@plos.org.

You will receive an invoice from PLOS for your publication fee after your manuscript has reached the completed accept phase. If you receive an email requesting payment before acceptance or for any other service, this may be a phishing scheme. Learn how to identify phishing emails and protect your accounts at https://explore.plos.org/phishing.

If we can help with anything else, please email us at customercare@plos.org.

Thank you for submitting your work to PLOS ONE and supporting open access.

Kind regards,

PLOS ONE Editorial Office Staff

on behalf of

Dr. Paul Obeng

Academic Editor

PLOS One
